# Darunavir pharmacokinetics throughout pregnancy and postpartum

**DOI:** 10.7448/IAS.17.4.19485

**Published:** 2014-11-02

**Authors:** John Lambert, Valerie Jackson, Laura Else, Mairead Lawless, Grainne McDonald, Dave Le Blanc, Anjali Patel, Kelly Stephens, Saye Khoo

**Affiliations:** 1 Infectious Diseases, The Rotunda Hospital, Dublin, Ireland; 2 Bioanalytical Facility, Royal Liverpool University Hospital, Liverpool, UK; 3 Department of Laboratory Medicine, The Rotunda Hospital, Dublin, Ireland; 4 Catherine McAuley Clinical Research Centre, Mater Misericordiae University Hospital, Dublin, Ireland; 5 Molecular and Clinical Pharmacology, Royal Liverpool University Hospital, Liverpool, UK

## Abstract

**Introduction:**

Antiretroviral therapy is recommended during pregnancy for prevention of mother-to-child transmission (MTCT) of HIV. Physiological changes during pregnancy are known to affect the pharmacokinetics (PK) of protease inhibitors (PIs), leading to lower exposures in pregnant women. Here we examine the PK of DRV/r 800/100 mg once daily (OD) over the course of pregnancy and postpartum (PP).

**Material and Methods:**

In this prospective open-labelled study, HIV-positive pregnant women receiving darunavir/ritonavir as part of their routine maternity care were enrolled. DRV plasma trough concentrations [DRV] were determined in the first (T1) and/or second (T2) and/or third (T3) trimester and PP using a validated HPLC-MS/MS methodology (Lab21, Cambridge UK). Where possible paired maternal and cord blood samples were taken at delivery.

**Results:**

To date 20 women (12 black African, 8 Caucasian) have been enrolled. Median (range) baseline CD4 count was 338 cells/µL (108–715), and median baseline plasma viral load was 555 copies/mL (<40–8,188,943). All but 2 women were virally suppressed at time of delivery (114 and 176 copies/mL; 1 sub-therapeutic at T3) and median CD4 count was 410 cells/µL (92–947). There were 20 live births, all term deliveries and there were no cases of MTCT. [DRV] (geometric mean; 95% CI) was 3790 ng/mL at T1 (*n*=1); 1288 ng/mL (663–1913) at T2 (*n*=9); 1086 ng/mL (745–1428) at T3 (*n*=18, 1 undetectable) and 2324 ng/mL (1369–3279) at PP (*n*=14, 1 undetectable). There was no significant difference in [DRV] between T2 and PP (*p*=0.158); however, there was between T3 and PP (*p*=0.021). Nineteen of twenty (95%) and 16 of 20 (80%) women achieved [DRV] above the estimated MEC for WT (55 ng/mL) and PI resistant HIV-1 (550 ng/mL) throughout pregnancy. Maternal and cord [DRV] were available for 10 mother–baby pairs. Mean maternal [DRV] at delivery was 2235 ng/mL (±1557 ng/mL), while mean cord [DRV] was 337 ng/mL (±217 ng/mL). The median cord to maternal blood ratio (C/M) was 0.11 (0.06–0.49).

**Conclusions:**

In most cases examined, DRV/r 800/100 mg once daily was effective at achieving adequate therapeutic drug levels (>550 ng/ml) during pregnancy. However, reduced DRV plasma concentrations in the second/third trimesters highlights the need for TDM in this population and warrants further study of pregnancy-associated changes in DRV pharmacokinetics. The low C/M ratios reported here are consistent with previous reports [1] and suggest low transplacental transfer of DRV.
